# Reprogramming of Tomato Leaf Metabolome by the Activity of Heat Stress Transcription Factor HsfB1

**DOI:** 10.3389/fpls.2020.610599

**Published:** 2020-12-23

**Authors:** Marine Josephine Paupière, Yury Tikunov, Enrico Schleiff, Arnaud Bovy, Sotirios Fragkostefanakis

**Affiliations:** ^1^Plant Breeding, Wageningen University, Wageningen, Netherlands; ^2^Faculty of Biological Sciences, Molecular Cell Biology of Plants, Goethe University, Frankfurt, Germany; ^3^Buchmann Institute for Molecular Life Sciences (BMLS), Goethe University, Frankfurt, Germany; ^4^Frankfurt Institute of Advanced Studies (FIAS), Frankfurt, Germany

**Keywords:** flavonoid, heat stress, metabolomics, *Solanum lycopersicum*, transcription, phenylpropanoid

## Abstract

Plants respond to high temperatures with global changes of the transcriptome, proteome, and metabolome. Heat stress transcription factors (Hsfs) are the core regulators of transcriptome responses as they control the reprogramming of expression of hundreds of genes. The thermotolerance-related function of Hsfs is mainly based on the regulation of many heat shock proteins (HSPs). Instead, the Hsf-dependent reprogramming of metabolic pathways and their contribution to thermotolerance are not well described. In tomato (*Solanum lycopersicum*), manipulation of HsfB1, either by suppression or overexpression (OE) leads to enhanced thermotolerance and coincides with distinct profile of metabolic routes based on a metabolome profiling of wild-type (WT) and HsfB1 transgenic plants. Leaves of HsfB1 knock-down plants show an accumulation of metabolites with a positive effect on thermotolerance such as the sugars sucrose and glucose and the polyamine putrescine. OE of HsfB1 leads to the accumulation of products of the phenylpropanoid and flavonoid pathways, including several caffeoyl quinic acid isomers. The latter is due to the enhanced transcription of genes coding key enzymes in both pathways, in some cases in both non-stressed and stressed plants. Our results show that beyond the control of the expression of Hsfs and HSPs, HsfB1 has a wider activity range by regulating important metabolic pathways providing an important link between stress response and physiological tomato development.

## Introduction

Plants trigger a cascade of reactions to acclimate to and survive heat stress (HS) conditions (Zhu, 2016). The transcriptional reprogramming marked by the induction or repression of hundreds of genes is central for HS response (HSR) (Ohama et al., 2017). Changes in mRNA levels lead to alterations in the proteome and consequently in the metabolome landscape. These changes are required for the accumulation of proteins and metabolites with protective functions (Paupière et al., 2014; Chaturvedi et al., 2016; Echevarría-Zomeño et al., 2016). For instance, antioxidants (e.g., flavonoids) are required for ROS detoxification and osmolytes (e.g., sugars and proline) contribute to the cellular water content adjustment and protection of membrane integrity (Rizhsky et al., 2004; Guy et al., 2008). The accumulation of specific metabolites prior to HS can also affect the molecular HSR such as γ-aminobutyric acid (GABA) which has a thermoprotective role (Taylor et al., 2010).

Heat stress transcription factors (Hsf) control the majority of transcriptional alterations that occur in heat stressed cells including many heat shock proteins (Hsp) (Scharf et al., 2012). In addition, Hsfs regulate genes coding for proteins with various functions (Brooks et al., 2014; Fragkostefanakis et al., 2016, 2018; Huang et al., 2016; Tian et al., 2019). Some of the genes required for metabolite production are regulated by Hsfs such as the Hsf1b-controlled galactinol synthase (GolS1) involved in raffinose biosynthesis in *Arabidopsis thaliana* (Panikulangara et al., 2004). HsfA2 knock-down in tomato anthers altered the abundance of several metabolites including the suppression of putrescine and GABA accumulation (Fragkostefanakis et al., 2016).

Tomato HsfB1 acts as repressor of several Hsfs, but also as co-activator of the master regulator HsfA1a (Bharti et al., 2004; Fragkostefanakis et al., 2018). HsfB1 suppression leads to the accumulation of several Hsfs and consequently to an increased leaf photosynthetic rate in response to HS (Fragkostefanakis et al., 2018). Enhanced expression of HsfB1 has a positive impact on thermotolerance as well, most likely due to an enhanced co-activation of HsfA1a, which leads to the synthesis of Hsps (Fragkostefanakis et al., 2018). However, the induction of HS-responsive genes and proteins already under non-stress conditions in HsfB1 overexpression (OE) lines leads to growth and developmental defects including reduced growth and flower abortion. Therefore, both HsfB1 knockdown and OE lines improve thermotolerance, although mediated through different regulatory routes. The importance of HsfB1 for the metabolome status of tomato plants under non-stress and HS conditions has not been explored, although the significant proteome alterations in HsfB1 OE and knock-down plants point to this direction. Here, comparison of the metabolome profile of wild-type (WT) and transgenic plants before and after HS allowed us to identify distinct and common pathways potentially related to enhanced thermotolerance in these lines and provide insights into new levels of regulation of HS resilience by the important HsfB1. Our results uncover an important link between the activity of this particular Hsf and HS-dependent metabolome changes.

## Materials and Methods

### Plant Material and Treatments

Wild type *Solanum lycopersicum* cv. Moneymaker and homozygous plants of T4 transgenic lines overexpressing HsfB1 (B1OE-5, -41, and -45), antisense (B1AS-18, -21), and co-suppression (B1CS-39; Fragkostefanakis et al., 2018) were cultivated in greenhouse in pots with soil/perlite (3:1 ratio) under a 16/8 h day/night cycle (25°C day/20°C night) with light intensity at canopy level of approximately 120 μmol m^–2^ s^–1^. Eight-week-old plants were transferred to a CLF Plant Climatics chamber (120 μmol m^–2^ s^–1^ light intensity), exposed to HS (1 h, 38°C) and then allowed to recover at 25°C after 1.5 h for gene expression analysis or 2 and 6 h for metabolome analysis. The stress was applied at 12.00 am and included a 30 min gradual increase while a 30 min gradual decreased from 38–25°C took place in the recovery phase which took place in the same chamber that the stress was applied. Control plants (25°C) were harvested prior to HS. The two youngest fully emerged leaves were pooled from five independent plants for each condition. The experiment was repeated five times for metabolome profiling and independently three for gene expression analysis (biological replicates), from different set of plants at different days.

### Extraction of Leaf Metabolites

Extraction of polar and semi-polar metabolites was carried out at 20°C using water:methanol:choloroform separation (Carreno-Quintero et al., 2012; Wahyuni et al., 2013). 700 μL of 100% methanol and 300 μL of distilled water were added to 100 mg of frozen homogenized leaf material followed by sonication (20 min) and centrifugation (17,000 × *g*, 10 min). Supernatant was divided into two portions A and B for GC-TOF-MS and LC-QTOF-MS analyses, respectively. 300 μL of portion (A) was supplemented with 600 μL distilled water and 400 μL chloroform followed by shaking (5 min) and centrifugation (17,000 × *g*, 10 min). Supernatant (40 μL) was transferred to a chromatographic glass vial (1.5 mL) with a glass insert and dried overnight in a centrifugal evaporator, the vials were then crimp capped under argon flow and subjected to a GC-TOF-MS analysis. Portion B was filtered through 0.2 μm polytetrafluoroethylene (PTFE) filter into 1.5 mL chromatographic glass vials, crimp capped and subjected to a LC-QTOF-MS analysis.

### Metabolomic Profiling

Semi-polar metabolites (extract B) were profiled by LC-QTOF-MS (Tikunov et al., 2010) using a Waters Alliance 2795 HT HPLC system equipped with a Luna C18(2) precolumn (2.0 mm × 4 mm) and an analytical column (2.0 mm × 150 mm, 100 Å, particle size 3 μm; Phenomenex) connected to an Ultima V4.00.00 QTOF mass spectrometer (Waters, MS Technologies). Degassed formic acid:ultrapure water (1:1,000, v/v; eluent A) and formic acid:acetonitrile (1:1,000, v/v; eluent B) were pumped at 190 μL min^–1^ with a linear gradient from 5–35% eluent B (45 min), followed by washing and re-equilibration. The column and sample were kept at 40 and 20°C, respectively. Ionization was performed using an electrospray ionization source, and masses were detected in negative mode (collision energy: 10 eV; m/z range: 100–1,500). Data were recorded with MassLynx 4.0 software (Waters).

Polar primary metabolites (extract A) were analyzed with an Optic 3 HP-injector (ATAS) and an Agilent 6890 gas chromatograph coupled to a Pegasus III TOF-MS (Leco; Carreno-Quintero et al., 2012). For that 12.5 μL *O*-methylhydroxylamine hydrochloride (20 mg mL^–1^ pyridine) was added to the dried samples and incubated (30 min, 40°C). Samples were derivatized with 17.5 μL *N*-methyl-*N*-trimethylsilyltrifluoroacetamide for 60 min. Two microliters were introduced into the injector at 70°C with a split ratio of 20 mL min^–1^ followed by heating to 240°C (6°C s^–1^). Chromatographic separation was performed using a VF-5ms capillary column (Varian; 30 m × 0.25 mm × 0.25 μm) including a 10-m guardian column (carrier-gas: helium; flow rate: 1 mL min^–1^) with a temperature ramp [2 min at 70°C, increase to 310°C (10°C min^–1^), 5 min at 310°C; transfer line temperature: 270°C]. The column effluent was ionized by electron impact at 70 eV. Mass spectra were acquired at 20 scans s^–1^ within m/z range of 50–600, 200°C source temperature, 295 s solvent delay, and 1,700 V detector voltage. The data were recorded with ChromaTOF software 2.0.

### Metabolite Data Processing

Data were processed in MetAlign software^1^ to correct for the baseline, eliminate noise, and perform mass spectral alignment of chromatograms (Tikunov et al., 2005; De Vos et al., 2007). Values lower than the detection threshold, 50 for GC-TOF-MS and 20 for LC-QTOF-MS, in more than three leaf samples once the estimated noise signal was subtracted from the estimated peak signal were omitted. Signals with m/z values <85 Da were considered non-specific and deleted. Compound mass spectra and quantitative ions were extracted from the modified MetAlign outputs as previously described (Tikunov et al., 2012) by MsClust (see text footnote 1). Only primary and secondary metabolite quantitatively present in all replicates of at least one of the experimental conditions were used. If a quantitative ion automatically selected by MSClust showed saturation of the MS detector, this ion was replaced by its second or third isotopic ion. Mass spectra produced by MSClust was used for compound annotation.

### Metabolite Annotation

Annotation of semi-polar ions was performed by comparing the detected mass spectra and retention time of compounds with the MoTo metabolite database (Moco et al., 2006) and metabolite online databases (Dictionary of Natural Products^2^; METLIN^3^) according to the Metabolomics Standards Initiative requirements (Sumner et al., 2007). Annotation of GC-TOF-MS metabolites was done by automatic mass spectra matching to the Golm metabolome database^4^ using MS Search v2.0 software of the National Institute of Standards and Technology (NIST). Three compound annotation hits were derived. Compound annotation was based on match factor and the difference of retention index between the library entries and the data. The Identified compounds were annotated level 1 when NMR was performed on the corresponding compounds of the library; level 2 was assigned when an analytical standard was used to annotate the compounds from the library or when a tandem mass spectrometry was performed; and level 3 was assigned when compounds were annotated based on their mass based on existing literature (Moco et al., 2006; Iijima et al., 2008; Gómez-Romero et al., 2010; Tikunov et al., 2010, 2013; Vallverdú-Queralt et al., 2010; Roldan et al., 2014).

### Statistical Analysis

The metabolite abundance values were normalized by fresh weight. The statistical analyses were performed with the IBM-SPSS statistic software package 20^5^. The univariate analysis was performed with one-way ANOVA and a Duncan’s *post hoc* test (*p* < 0.05). The multivariate analysis was performed by principal component analysis on log2 transformed and mean centered values with GeneMaths XT (version 2.12). Paired *t*-tests were used for comparison of gene expression in WT and transgenic lines under same conditions.

### Transcript Analysis

Total RNA was extracted (E.Z.N.A. Plant RNA Kit; Omega BioTek, Norcross, GA, United States) from tomato leaves and cDNA was synthesized with Revert Aid reverse transcriptase (Thermo Scientific) following the manufacturer’s protocols. Expression of genes was determined using quantitative real-time PCR (qRT-PCR) using gene-specific oligonucleotides (Table 1), PowerUp SYBR Green Mastermix (ThermoFischer, Carlsbad, CA, United States) and cDNA template, under conditions previously described (Fragkostefanakis et al., 2018). Data were analyzed by 2^–ΔΔCt^ method (Livak and Schmittgen, 2001) using *EF1*α (Solyc06g005060) as reference gene.

**TABLE 1:** List of oligonucleotides used for qRT-PCR analysis.

**Gene**	**Accession number**	**Forward oligonucleotide (5′-3′)**	**Reverse oligonucleotide (5′-3′)**
HCT	Solyc03g117600	TATGGCACGAGGACTCACTC	ATCACCAGCAACAGCAACAG
C3H1	Solyc01g096670	CTGTTGCTCGCGACCTCAAG	ACCTAATTGTGCCCCTGGAC
C3H2	Solyc10g078240	CTGTGGCTCGTGATCCAGCA	TGACCCAACATAGAAGTGACCA
HQT	Solyc07g005760	AGTCCAGTACCACGACCATG	TGGGCTGCGAGGATTTCATA
CHS1	Solyc09g091510	GTTCCGTGGACCCAGTGAAT	AAAAGGGCTTGGCCTACCA
CHS2	Solyc05g053550	GGCCGGCGATTCTAGATCA	TTTCGGGCTTTAGGCTCAGTT
CHI-1	Solyc05g010320	GAAGCAGTGCTCGATTCCATAAT	GTTTTTCACAAACCAACAGTTCTGAT
CHI-L	Solyc05g052240	GCGATAGAAGGTAAGGA	AGCCAAAGAAGAAATAGTTGT
F3H	Solyc02g083860	CACACCGATCCAGGAACCAT	GCCCACCAACTTGGTCTTGTA
F3′H	Solyc03g115220	GCACCACGAATGCACTTGC	CGTTAGTACCGTCGGCGAAT
FLS	Solyc11g013110	GAGCATGAAGTTGGGCCAAT	TGGTGGGTTGGCCTCATTAA
3GT	Solyc07g052640	CGAACGACGAAACACTGTTGA	TGCAGCATAGATGGCATTGG
PAL1	Solyc10g086180	GACTGCAGGAAGGAATCCAA	TTAAGCCCAAGGAGTTCACG
PAL2	Solyc05g056170	GATCGTTACGCGCTTAGGAC	CCTATTGGTGTCCCTTGGAA
C4L	Solyc03g097030	CAATGACGATGAAGCCACTG	GCAGCATCTGCAATATCTGG
C4H	Solyc05g047530	GCTGTTGCTGGTGGTAAGGT	GCTTTGCAATGTGCAAGCTA
EF1a	Solyc06g005060	GGAACTTGAGAAGGAGCCTAAG	CAACACCCACAGCAACAGTTT

*Oligonucleotides were designed using PRIMER3 (bioinfo.ut.ee/primer3-0.4.0/).*

### Statistical Analysis

The abundance values of both platforms were normalized by fresh weight and then log2 transformed. All the statistical analyses were performed with the IBM SPSS statistic software package 20 (see text footnote 5). The univariate analysis was performed with a one-way ANOVA for each compound followed by Duncan’s *post hoc* test (*p* < 0.05). The multivariate analysis was performed with a principal component analysis on log2 transformed and mean centered value with GeneMaths XT version 2.12 software.

## Results

### Profile of Primary and Secondary Metabolites in Tomato Leaves After HS

**FIGURE 1:**
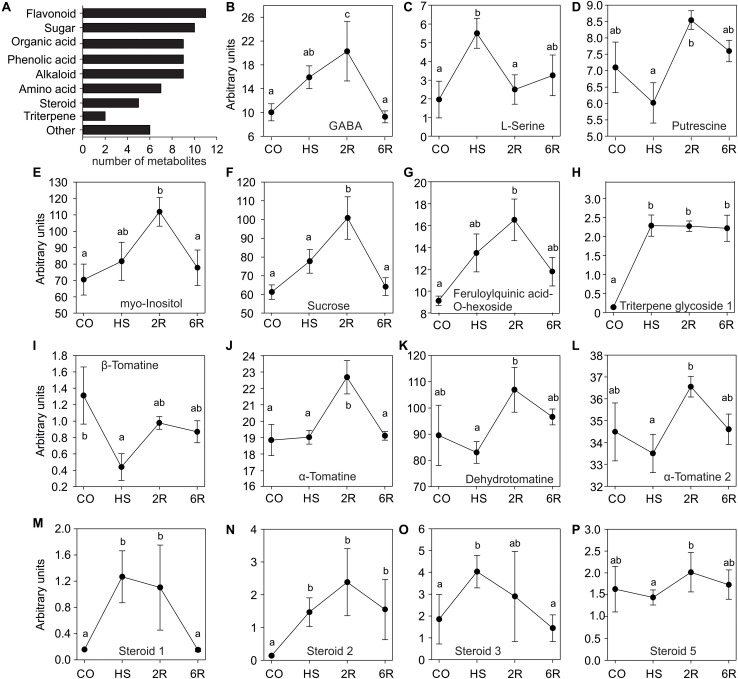
Metabolite profile of wild-type tomato leaves exposed to HS. **(A)** Categories of metabolites identified in this study. **(B–F)** Primary and **(G–P)** secondary metabolite levels in leaves of plants exposed to 38°C for 1 h (HS), and then allowed to recover for 2 (R2) or 6 h (R6), or kept for the same time at 25°C as control sample (CO). Values are MS detector responses – arbitrary units and represent the average of four or five independent biological replicates ± SE. Letters denote statistically significant differences (*p* < 0.05) based on one-way ANOVA and a *post hoc* Duncan test.

To study the impact of a HS treatment on the tomato leaf metabolome, GC-TOF-MS and LC-QTOF-MS analyses were used to determine the qualitative and quantitative composition of primary and secondary metabolites, respectively. Young leaves of eight-week old WT *Solanum lycopersicum* cv. Moneymaker were harvested under control temperature (CO, 25°C), after an exposure at 38°C for 1 h (HS), or following a 2 (2R) or 6 h (6R) recovery from HS at 25°C. In total 37 polar metabolites were detected of which 27 were annotated including amino acids, sugars, organic acids, amines, and phenolic acids (Figure 1A). In addition, 66 semi-polar metabolites were detected of which 41 were annotated including alkaloids, flavonoids, steroids, phenolic acids, organic acids, and triterpenes (Figure 1A and Supplementary Datasets 1, 2).

The profiles of 15 annotated metabolites revealed a significant change in abundance among the samples. Only the alkaloid (β-tomatine) shows a significant reduction upon HS when compared to the control (Figure 1I), while all others exhibited an accumulation either directly after HS or during the recovery phase. Serine (Figure 1C), a triterpene glycoside (Figure 1H), and three unknown steroid-like compound (Figures 1N–P) accumulated significantly after 1 h of HS. GABA, myo-inositol, sucrose, a-tomatine, and feruloylquinic acid-*O*-hexoside significantly accumulated after 2 h of recovery from stress and then returned to control levels (Figures 1B,E,F,G,K). Putrescine, dehydrotomatine and another unknown steroid-like compound showed no significant alteration upon 1 h of HS, but a transient increase after 2 h of recovery when compared to HS sample (Figures 1D,L,P). Therefore, despite the short stress treatment, we observed a dynamic metabolome alteration during HS and recovery.

### HsfB1 Is Important for the Metabolite Status in Tomato Leaves in Response to HS

**FIGURE 2:**
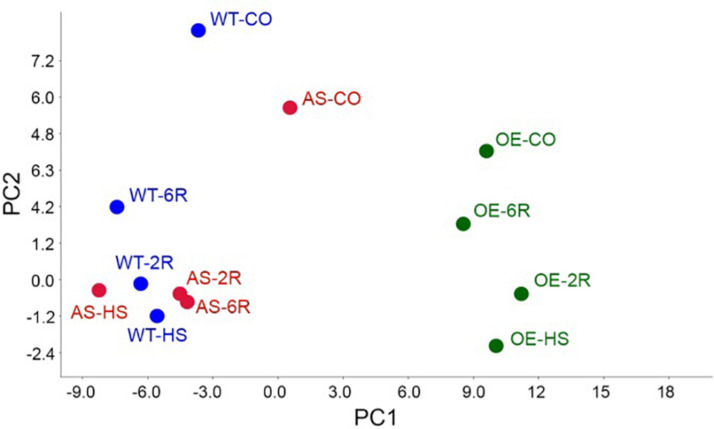
Principal component analysis (PCA). PCA of samples integrating both primary and secondary metabolites with the components 1 (PC1, 67%) and 2 (PC2, 12%) in WT, B1OE and B1AS lines in control (CO) and heat stressed (HS) leaves as well as after 2 (2R) or 6 h (6R) of recovery.

We performed the same analysis on one representative HsfB1 OE (B1OE-5) and one AS (B1AS-21) lines to study the impact of HsfB1 activity on the tomato leaf metabolome. The multivariate analysis including both primary and secondary metabolites showed that most of the variation of the metabolome (PC1 = 67% of the variation) could be attributed to the genetic background. A clear distinction between B1OE samples on the one hand and the WT and B1AS samples on the other hand was observed, independent of the treatment (Figure 2). PC2 (12% of variation) separated control from heat stressed samples for all genotypes (Figure 2). The variation of the metabolome between control and 6 h recovery state (6R) was the smallest in B1OE and the largest in B1AS samples (Figure 2). In the latter PC2 was comparable for HS, 2 h recovery (2R) and 6R samples, while PC2 for WT and B1OE reflected the attenuation of HS and the tendency to recover back to the control status. These results suggest that the activity of HsfB1 may facilitate in the return of the metabolome profile after HS to control levels.

**FIGURE 3:**
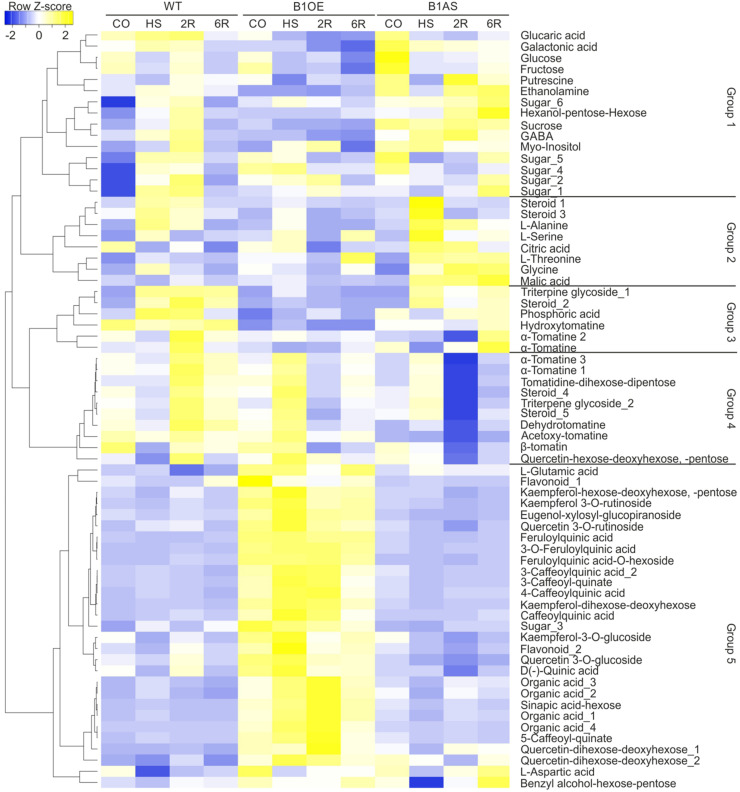
Effect of HsfB1 overexpression or suppression on abundance of metabolites in tomato leaves exposed to heat stress. Annotated metabolites were grouped based on their profile in all samples and genotypes using complete linkage clustering method based on Pearson distance measurement. The dendrogram showing similarity in metabolite profiles was constructed by complete linkage clustering and distances were calculated by Pearson index. Metabolite levels are depicted as heat map constructed using heatmapper (Babicki et al., 2016). Annotated metabolites and groups are depicted on the right.

The similarity of the profiles of the 68 annotated metabolites was examined by Pearson distance determination and metabolites were grouped based on complete linkage clustering method using Heatmapper (Babicki et al., 2016). This analysis yielded five major groups (Figure 3). Groups 1, 2, and 3 contained metabolites that have a diverse profile but have in general moderately or significantly lower levels in B1OE leaves, especially either after 2 or 6 h of recovery (2R, 6R). GABA and steroid_3 levels were significantly reduced in B1OE leaves in HS, 2R, and 6R samples when compared to WT, while, sucrose and glucose content was significantly reduced in 2R and 6R samples of B1OE leaves, respectively.

In group 1 several metabolites were significantly more abundant in B1AS control leaves when compared to WT, including glucose, sucrose, two sugar isoforms, putrescine, and ethanolamine (Figure 3). Metabolites of group 2 such as citric acid, threonine, and malic acid were more abundant in B1AS during HS. In group 3, a triterpene glycoside and an α-tomatine were less abundant in B1AS 2R sample when compared to WT corresponding sample. Group 4 contained metabolites which did not show any change upon HS in any of the genotypes, but all of them having significantly reduced levels in B1AS leaves after 2 and 6 h of recovery when compared to WT and B1OE samples. These metabolites included mainly glycoalkaloids, steroids, and triterpenes. Group 5 was comprised of 29 compounds which show constitutively enhanced levels in all B1OE samples when compared to WT or B1AS. The majority of these metabolites are flavonoids, phenolic compounds, and organic acids. In conclusion, silencing and OE of HsfB1 had a different effect on the metabolic profile of tomato leaves in different stages of the HSR.

### Analysis of Transcript Levels of Flavonoid Pathway Genes

**FIGURE 4:**
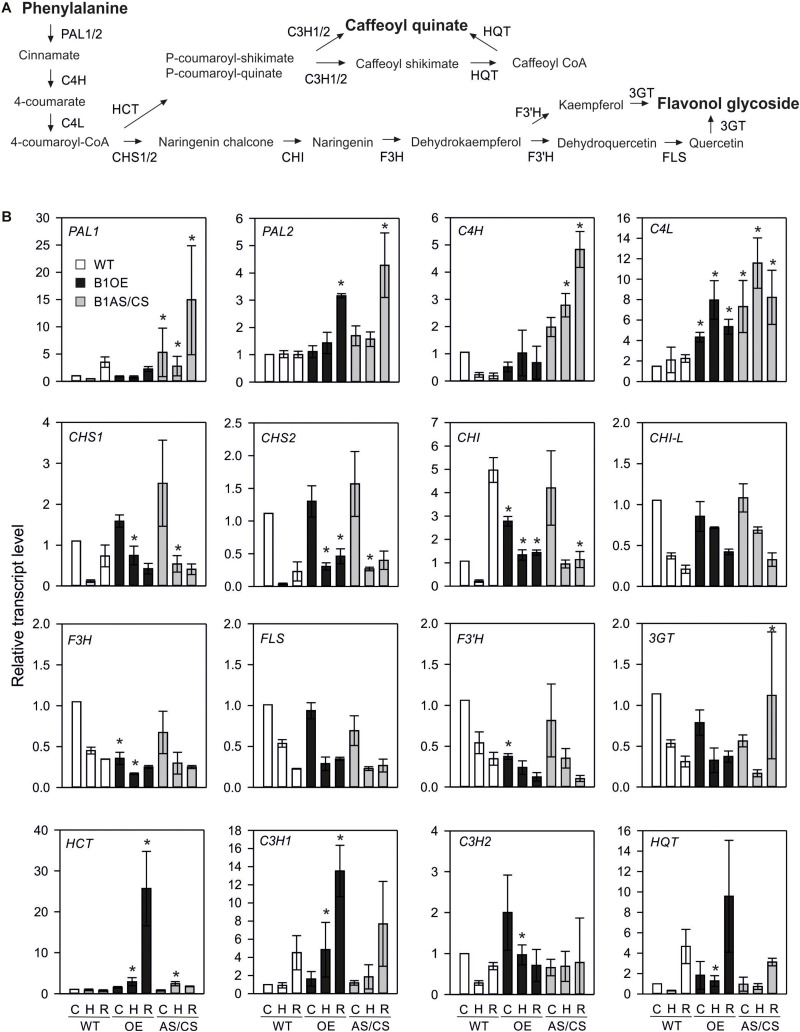
Effect of HsfB1 overexpression and suppression on transcript levels of phenylalanine pathway **(A)**. Transcript levels **(B)** were determined by qRT-PCR and normalized to wild-type control sample for each gene. Values are the average for three independent OE lines (B1OE-5, -41, -46), and three independent suppression lines (B1AS-18, B1AS-21, B1CS-39), ± SE. Transcript levels are expressed relative to WT control sample. Asterisk denotes statistically significant difference (*p* < 0.05) when compared to WT for the same temperature, based on paired *t*-test. Conditions: C, control; H, heat stress; R, recovery. Genotypes: WT, wild-type; OE, overexpression; AS/CS, antisense/co-suppression.

The strong constitutive accumulation of flavonoids in HsfB1 OE line prompted the investigation of the expression profile of genes of the corresponding flavonoid pathway in all lines. Here, in addition to B1OE-5 and B1AS-21 used for metabolome analysis, two more independent transgenic HsfB1 antisense lines, one AS (B1AS-18) and one co-suppression (B1CS-39) (both further denoted as AS/CS) and two OE lines (B1OE-41 and B1OE-45) were used and compared to WT (Fragkostefanakis et al., 2018). Control and HS samples were treated as described for the metabolome analysis, but recovery samples were harvested 1.5 h after they were returned to 25°C, supposing that transcriptional control precedes regulation at protein and metabolite levels. The expression of sixteen genes of the phenylpropanoid/flavonoid pathway (Zhang et al., 2015; Figure 4A) was studied by qRT-PCR (Figure 4B).

Phenylalanine to cinnamate conversion (the first reaction in the flavonoid pathway) is catalyzed by phenylalanine ammonia lyase (PAL). In WT plants, the abundance of *PAL1* transcript was reduced during HS, but enhanced during recovery to levels higher than observed in control sample (Figure 4B). In contrast, *PAL2* was not altered in WT in response to HS (Figure 4B). *PAL1* was enhanced in control leaves, HS and recovering leaves of AS/CS, but not of the OE lines, while *PAL2* showed increased levels in recovering leaves of both AS/CS and OE lines when compared to WT.

Cinnamate 4-hydroxylase (C4H) gene catalyzing the conversion of cinnamate to 4-coumarate was reduced in WT leaves after HS treatment. However, *C4H* was enhanced in HS and recovering leaves of HsfB1 AS lines when compared to WT. The transcripts of the gene coding for cinnamate 4-hydroxylase (C4L) catalyzing the further conversion of 4-coumarate to 4-coumaroyl-Co-A were enhanced in both OE and AS/CS lines compared to WT.

4-coumaroyl-Co-A can be routed either to lignin biosynthesis by convertion to caffeoyl quinate or to the flavonol biosynthesis pathway by conversion to naringenin chalcone, which is mediated by the chalcone synthase enzyme (CHS). Naringenin chalcone is then further converted to naringenin and dehydrokaempferol by chalcone isomerase (CHI) and the flavanone-3-hydroxylase (F3H), respectively. Finally, the activity of the flavonol synthase (FLS), the flavonoid-3′-hydroxylase (F3′H) and the flavonoid-3-*O*-glucosyltransferase (3GT) is required for the synthesis of flavonols and their glycosides. All eight tested genes, were downregulated during and/or after HS treatment in WT, AS/CS and OE plants. With the exception of *CHI-L* and *FLS*, all genes were affected by HsfB1 OE or knock-down.

Under non-stress conditions OE of HsfB1 led to higher levels of *CHI*, but reduced levels of *F3H* and *F3*′*H*. AS/CS of HsfB1 had no effect on these genes prior to HS application. After 1 h of HS, an increased transcript abundance of *CHS1*, *CHS2*, and *CHI* in comparison to WT was observed, while *F3H* expression was decreased. *CHS1* and *CHS2* were also more abundant in the HsfB1 AS/CS lines. After 1.5 h recovery, *CHS2* remained at the higher level in OE when compared to WT, while the transcript level of the different *CHI* genes were lower than WT in both, AS/CS and OE lines. *3GT* was abundant upon recovery of AS/CS lines. Summarizing, both OE and AS/CS of HsfB1 enhanced the levels of *CHS* genes after HS and recovery, while transcript levels of *CHI* were suppressed during recovery. In addition, OE of HsfB1 seems to have a negative impact on the expression levels of *F3H* and *F3*′*H.*

The integration of 4-coumaroyl-Co-A into the caffeoyl quinic acid biosynthesis pathway starts with the production of *p*-coumaroyl-shikimate or *p*-coumaroyl-quinate by the multifunctional enzyme hydroxycinnamoyl-CoA shikimate/quinate transferase (HCT). Depending on the precursor, the *p*-coumaroyl ester 3-hydroxylase encoded by the two genes C3H1 and C3H2 either directly produces caffeoyl-quinate or caffeoyl shikimate. The latter is converted to caffeoyl-quinate by the action of the HCT and the hydroxycinnamoyl-CoA quinate transferase (HQT). While *HCT* and *C3H1* showed steady state levels after 1 h HS treatment in WT leaves, *C3H2* and *HQT* were reduced. Following HS, *C3H1*, *C3H2*, and *HQT* transcript levels were enhanced after 1.5 h recovery at even higher levels than the control WT sample.

All four genes showed enhanced transcript levels in the OE lines in HS and both *C3H* genes in recovery samples when compared to WT. In contrast, only *HCT* showed increased levels after 1 h of HS in the HsfB1 AC/CS lines. Thus, the branch of caffeoyl quinate synthesis seems to be enhanced by HsfB1 OE.

## Discussion

Exposure to high temperatures leads to major metabolic alterations which support protection and maintenance of housekeeping cellular functions. Out of 68 annotated compounds we observed significant changes in the abundance of 15 metabolites upon a HS treatment in tomato leaves of WT plants (Figure 1). While β-tomatine levels were reduced after HS treatment, ten compounds had increased levels either directly after the HS treatment or upon recovery when compared to the control leaves (Figure 1). Among the accumulated metabolites we detected many that have been previously shown to have a positive impact on thermotolerance such as GABA, sugars, steroids, and several phenylpropanoid compounds and flavonoids (Commisso et al., 2016; Liu et al., 2019). Alterations in the profile of metabolites indicate either a variance in thermosensitivity levels of key enzymes and/or a variation in the levels of regulation of the relevant genes and proteins.

HsfB1 is minimally expressed in non-stressed leaves and therefore its suppression has a lower impact on the variation of metabolome when compared to the effect of the OE (Figure 2). These results are in agreement with the proteome variation in HsfB1 transgenic lines under similar conditions (Fragkostefanakis et al., 2018). After HS, a recovery of the metabolome back to control levels is observed (Figure 2). Thus, protective metabolites are either degraded or used for further processing as observed e.g., for serine or steroid_1 (Figure 1). In B1AS leaves the recovery at metabolite level is strongly reduced (Figure 2). This holds especially true for metabolites of group 4 (Figure 3). The metabolome profile of B1OE line is in general different from that of WT and B1AS suggesting a significant impact of HsfB1 accumulation already under control conditions (Figure 2; PC1). Interestingly, as shown by PC2, the metabolome composition of B1OE control is more related to the WT after 6 h of recovery than to WT control (Figure 2). Thus, our results highlight an impact on leaf metabolome by HsfB1 accumulation and the importance of the regulation of HsfB1 homeostatic levels particularly for the fine-tuned recovery of the cellular state after HS.

B1AS leaves accumulated several sugars including glucose and sucrose in control leaves (Figure 3). Sugars can enhance thermotolerance by acting as signaling molecules, osmolytes or contribute via their carbon to oligosaccharide pathways (Rizhsky et al., 2004; Serrano et al., 2019). In addition, the polyamine putrescine which acts as a radical scavenger (Asthir et al., 2012) also accumulated in non-stressed tomato leaves in B1AS line (Figure 3). Therefore, suppression of HsfB1 led to an activation of specific metabolic pathways upon HS which can potentially enhance thermotolerance. We assume that HsfB1 may act as repressor of genes coding for enzymes of these pathways.

HsfB1 OE led to a higher accumulation of compounds mainly belonging to the phenylpropanoid pathway. B1OE plants accumulate caffeoyl quinic acids and flavonoids, such as kaempferol- and quercetin glycosides, either already in non-stressed leaves or during HS (Figure 3). These compounds showed steady state levels in WT and B1AS leaves, indicating that HsfB1 OE shifts metabolism toward specific metabolic routes.

Phenolics have important antioxidant activity under stress conditions (Grace and Logan, 2000). We did not observe any significant changes in the levels of phenolics upon HS treatment in WT (Figure 3). Genes encoding for enzymes in this pathway were downregulated during HS, while the transcripts fully or partially returned to control levels already after 1.5 h of recovery, or even accumulated to higher levels in WT leaves (Figure 4). For several genes, HsfB1 OE or suppression led to either a weaker reduction in transcript levels or even an accumulation when compared to WT (Figure 4), suggesting that as proposed for Hsf-dependent networks like Hsps (Fragkostefanakis et al., 2018), a shift from the homeostatic levels of HsfB1, either up or down, can result in a similar molecular response. Nevertheless, HsfB1 OE resulted in significantly higher levels of the four genes involved in the production of several caffeoyl quinic acid (chlorogenic acid) isomers especially after HS (Figure 4), which were found to accumulate in the B1OE line (Figure 3). In contrast, *F3H* and *F3*′*H* which encode for enzymes required for synthesis of flavonoids are downregulated in B1OE leaves, while the respective flavonol glycosides are increased in these samples (Figures 3, 4). Therefore, while for the phenylpropanoid branch an apparent transcriptional regulation contributes to gene expression of biosynthetic enzymes, flavonoid pathway is not generally affected the same way. We assume that either an enhanced expression of a gene responsible for a rate-limiting step such as *CHI* may be sufficient to cause an accumulation of flavonoids, or the regulation of this pathway might occur at the posttranscriptional level, for example in a chaperone-dependent manner. We did not identify the corresponding proteins in our previous proteomics study (Fragkostefanakis et al., 2018) and thus we cannot fully explain the basis of such differences. The phenylpropanoid pathway is the source for lignin biosynthesis. An enhanced lignification, important for secondary growth can, at least in part, explain the stunted growth observed in B1OE plants (Fragkostefanakis et al., 2018).

While the impact of HS on the metabolome profile of plants is documented, currently, the integration of metabolic pathways into the central HSR mechanisms, mainly regulated by Hsfs, is not well understood. Here we show that suppression of HsfB1 affects different metabolic pathways compared to the OE. We propose that this cannot be fully dependent on the chaperone activity of HSPs, as we have previously shown that both suppression and OE of HsfB1 lead to enhanced HSP synthesis compared to WT. However, whether HsfB1 directly regulates relevant genes for the respective pathways needs to be experimentally addressed in the future. Nevertheless, our results suggest the diverse functions of Hsf-dependent networks for thermotolerance and expand the current chaperone-oriented models to highlight the importance of readjustment of metabolism to meet cellular and organismic needs for protection and recovery from stress.

## Data Availability Statement

The original contributions presented in the study are included in the article/Supplementary Material, further inquiries can be directed to the corresponding author/s.

## Author Contributions

MP performed the metabolome analysis with the assistance of YT. SF performed the stress treatments and the gene expression analysis. SF, AB, and ES conceptualized and headed the project. SF and ES wrote the manuscript with contribution from all authors. All authors have read and approved the manuscript.

## Conflict of Interest

The authors declare that the research was conducted in the absence of any commercial or financial relationships that could be construed as a potential conflict of interest.

## Footnotes

1 www.metalign.nl

2 http://dnp.chemnetbase.com

3 http://metlin.scripps.edu

4 http://gmd.mpimp-golm.mpg.de/

5 www.ibm.com

## Supplementary Material

Supplemental Dataset 1 Metabolites identified by GC-TOF-MS analysis.

Supplemental Dataset 2 Metabolites identified by LC-QTOF-MS analysis.
